# Doxorubicin-loaded gold nanorods: a multifunctional chemo-photothermal nanoplatform for cancer management

**DOI:** 10.3762/bjnano.12.24

**Published:** 2021-03-31

**Authors:** Uzma Azeem Awan, Abida Raza, Shaukat Ali, Rida Fatima Saeed, Nosheen Akhtar

**Affiliations:** 1 Department of Biological Sciences, National University of Medical Sciences (NUMS), Rawalpindi, Pakistan; 2 NILOP Nanomedicine Research Laboratories, National Institute of Lasers and Optronics College, (PIEAS), Islamabad, Pakistan; 3 Medical Toxicology Lab, Department of Zoology, Government College University Lahore, Lahore-54000, Pakistan

**Keywords:** chemotherapy, doxorubicin, gold nanorods, NIR laser, photothermal therapy

## Abstract

Two of the limitations associated with cancer treatment are the low efficacy and the high dose-related side effects of anticancer drugs. The purpose of the current study was to fabricate biocompatible multifunctional drug-loaded nanoscale moieties for co-therapy (chemo-photothermal therapy) with maximum efficacy and minimum side effects. Herein, we report in vitro anticancerous effects of doxorubicin (DOX) loaded on gold nanorods coated with the polyelectrolyte poly(sodium-4-styrenesulfonate) (PSS-GNRs) with and without NIR laser (808 nm, power density = 1.5 W/cm^2^ for 2 min) irradiation. The drug-loading capacity of PSS-GNRs was about 76% with a drug loading content of 3.2 mg DOX/mL. The cumulative DOX release significantly increased after laser exposure compared to non-irradiated samples (*p* < 0.05). The zeta potential values of GNRs, PSS-GNRs and DOX-PSS-GNRs were measured as 42 ± 0.1 mV, −40 ± 0.3 mV and 39.3 ± 0.6 mV, respectively. PSS-GNRs nanocomplexes were found to be biocompatible and showed higher photothermal stability. The DOX-conjugated nanocomplexes with NIR laser irradiation appear more efficient in cell inhibition (93%) than those without laser exposure (65%) and doxorubicin alone (84%). The IC_50_ values of PSS-GNRs-DOX and PSS-GNRs-DOX were measured as 7.99 and 3.12 µg/mL, respectively, with laser irradiation. Thus, a combinatorial approach based on chemotherapy and photothermal strategies appears to be a promising platform in cancer management.

## Introduction

Despite the enormous advances in medical research, cancer is still the second most common cause of death worldwide from which 9.6 million people died in 2018 [1]. Hepatocellular carcinoma (HCC) is one of the major types of liver cancer with high incidence of mortality [2]. Currently, there are a number of treatment modalities, including chemotherapy, immunotherapy, targeted therapy, irradiation, and surgery [3]. Among these, chemotherapy is the most commonly used method as most of the HCC patients are diagnosed at advanced stages and are not good candidates for liver transplantation or surgical resection [4–5]. However, the use of conventional chemotherapeutic agents in cancer treatment is limited due to several unwanted characteristics of poor solubility, broad bioavailability range, narrow therapeutic index, rapid elimination from systemic circulation, unselective site of action after oral/intravenous administration, and cytotoxic effects on normal tissues [6]. The anticancer drug doxorubicin (DOX) is extensively used in the management of different tumors [7] and exerts antitumor activity by interaction with DNA replication [8]. DOX-based chemotherapy is one of the main treatments for HCC but its efficacy is limited by pre-existing and acquired drug resistance due to long-term chemotherapy [9]. Also, high-dose regimens of DOX are associated with sever cardiotoxicity and bone marrow suppression. Different strategies were used to encapsulate the drug to minimize its side effects; however, this decreased the chemotherapeutic effectiveness [10]. Henceforth, new treatment modalities are urgently needed to kill cancerous cells without damaging normal cells or tissues. One approach is to selectively remove cancer cells using the advanced drug delivery systems. These carrier systems hold sufficient amounts of the drug with prolonged circulation time and sustained drug release at the tumor site [11].

Nanotechnology provides a means to overcome these hurdles as nanocarriers, which improve the pharmacological properties of free drugs, contribute to enhanced therapeutic efficacy in physiological environment [12]. Nanocarriers as multifunctional tumor targeting and therapeutic agents exhibit properties such as significant absorption or scattering in the visible and near-infrared (NIR) regions, tunable aspect ratio, biocompatibility, fluorescence properties, and the ease of biofunctionalization, which makes them ideal in biomedical applications [13]. Gold-based nanomaterials (i.e., nanospheres, nanorods, nanoshells, and nanocages) have great potential in photothermal cancer therapy due to plasmonic properties and the ease of biofunctionalization. Gold nanorods (GNRs) are more preferable than other gold nanomaterials because of their photothermal conversion efficiency. Better nanotherapeutics can be obtained by utilizing external stimuli, such as pH value, light, or ultrasound, to deliver the anti-cancerous drug into tumor tissue with spatial and temporal control [14]. Photothermal therapy (PTT) is an emerging minimally invasive cancer therapy. It can efficiently induce cytotoxicity by conversion of absorbed NIR light to heat. In cancer intervention, NIR-mediated photothermal therapy is gaining more attention due to the deep tissue penetration with minimal absorbance by healthy tissues [15–16]. Gold nanorods are potential delivery carriers for sustained drug release in response to an external stimulus [13]. Additionally, the NIR light-induced heat can improve the sensitivity of cancer cells towards chemotherapeutic agents by increasing blood vessel dilation and membrane permeability. These findings provide an incentive to combine photothermal therapy and chemotherapy for cancer treatments.

Regardless of the various beneficial properties, GNRs have limitations in clinical applications due to the cytotoxicity of the surfactant cetyltrimethylammonium bromide (CTAB), which acts as a template in the synthesis process of GNRs [17]. Different polymers can be used to coat GNRs to enhance their biocompatibility and dispersion at physiological pH values. The positive CTAB layer on the GNR surface facilitates electrostatic adsorption of anionic compounds, such as poly(sodium 4-styrenesulfonate) (PSS), which ultimately facilitates electrostatic interaction with cationic anticancerous drugs, such as DOX [18]. Advanced synergistic therapies, such as the combination of chemotherapy and photothermal therapy, have been applied to enhance the overall therapeutic efficacy [19]. This includes magnetic cores capped with gold nanorods, silica nanorattle gold shells, and DNA-based platforms loaded with GNRs and DOX [20–22].

Venkatesan et al. developed a DOX-loaded PSS-coated GNR nanoplatform via electrostatic interaction that selectively delivered DOX to target cells and effectively inhibited tumor growth in MCF-7 cells [18]. The killing effect of the DOX@PSSAuNRs was more pronounced at low concentrations (0.5–2 µg/mL) and higher cytotoxicity compared to free DOX was observed. However, no significant difference was reported at a higher concentration of 5 µg/mL. 68.5% and 62.4% of cells was killed by the DOX@PSS-Au NR conjugate and free DOX, respectively. To achieve significant cytotoxicity with the nanocomplex compared to free DOX, herein, we have used the same strategy as described in an earlier report [18] to design a multifunctional PSS-coated GNRs-based nano-platform that facilitate chemotherapy by delivering anticancerous drug at the site of action. DOX release with precise temporal and spatial control is triggered under local hyperthermic conditions induced by NIR laser irradiation. Heat from the GNR surface not only promotes drug delivery into the tumor, but also increases the drug toxicity to tumor cells by the hyperthermic effect. A significantly higher cell death rate was achieved in the tumor cells treated with chemo and photothermal co-therapy compared to the free drug. One of the major limitations associated with photothermal therapy is the usage of high laser powers for long time durations. We used a NIR laser power density of 1.5 W/cm^2^ for 2 min, which are a lower power density and a shorter irradiation time, respectively, compared to many previously reported studies. Liao et al. reported cell death at higher laser power (2.5 W/cm^2^) with longer exposure times [23]. Although, Chen and colleagues have reported cell death at a low laser power density of 1.8 W/cm^2^ but they used a high exposure time and a comparatively high drug concentration (20 µg/mL) [24]. We observed significant cell death at a lower laser power density using a shorter exposure time and a lower drug concentration. This will minimize thermotoxicity associated with laser exposure.

## Results and Discussion

### Synthesis of the DOX-loaded GNR nanocomplex

In the present study, DOX-conjugated GNRs and hyperthermia were employed as a treatment strategy for HCC cells. First, GNRs were synthesized according to the well-known seed-mediated growth method [25] with a slight modification reported in our previous work [26]. The uniform GNRs were synthesized with an aspect ratio of 4.3 (26 ± 2 nm in length and 6 ± 3 nm in width), by keeping pH value (pH 3) and temperature (*T* = 28 °C) constant.

**Figure 1:**
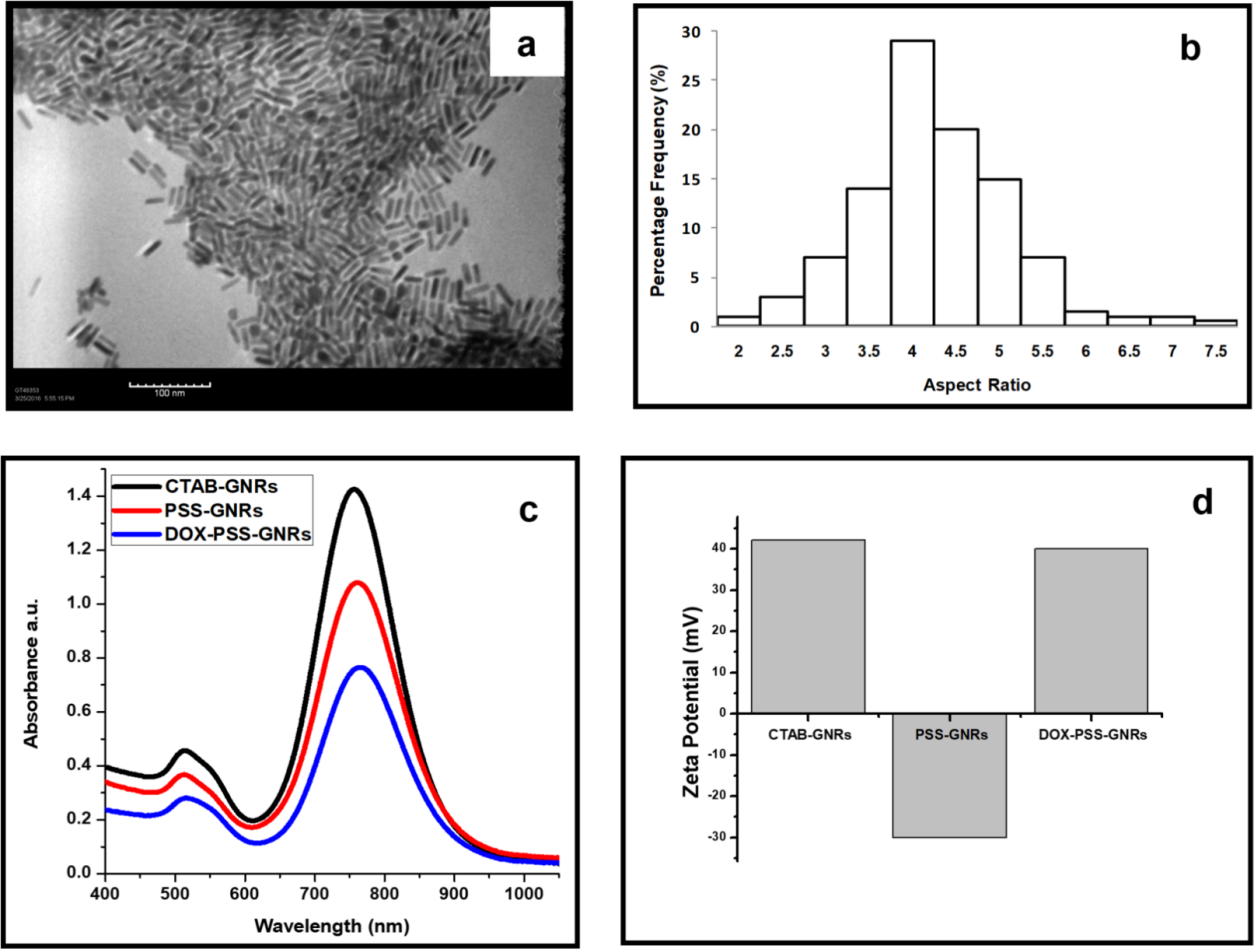
(a) TEM image of monodispersed GNRs. (b) Histogram showing the aspect ratio of GNRs. (c) UV–vis absorption spectrum of bare GNRs, PSS-coated GNRs and DOX-loaded PSS-GNRs. (d) Zeta potential of CTAB-coated GNRs, PSS-GNRs and DOX-PSS-GNRs.

The prepared GNR suspension has a surplus of cytotoxic CTAB, which was removed by repetitive cycles of centrifugation and re-dispersion. A CTAB bilayer remained non-covalently bound onto the GNRs surface to maintain the stability of the final product. The longitudinal localized plasmon resonance (LSPR) and the transverse plasmon resonance (TSPR) of the prepared GNRs were found to be 780 and 526 nm, respectively. TEM images display mono-dispersed rods with an aspect ratio of 4.2 (Figure 1a,b). GNRs could be potential candidates for photothermal therapy because their LSPR absorption band lies in the NIR region in which transmitted light caused no obvious damage to healthy tissues. Biocompatible GNRs were prepared through coating their surface with PSS. The LSPR peak of the PSS-coated GNRs was slightly redshifted to 783 nm (Figure 1c). The shift of the LSPR peak after PSS coating is due to the side-by-side assembly of the PSS-GNRs [27]. The surface charge of the GNRs changed from strongly positive (+42 mV, due to CTAB presence) to negative after PSS modification, which also confirmed the successful surface modification as described in previous reports [28].

Absorption spectra confirmed the successful loading of DOX on the PSS-coated GNRs (Figure 1c). The polyelectrolyte coating allowed the GNRs to easily interact with the surrounding environment. Consequently, the LSPR wavelength of the GNRs perceptively responded to the refractive index change caused by molecular adsorption. The conjugation of DOX onto the surface of the PSS-GNRs resulted in a redshift of the LSPR band, while the TSPR peak did not shift. The increased local refractive index around GNRs due to adsorption of DOX might lead to a stronger Columbic restoring force and a redshift of the LSPR peak [29]. The zeta potential of unrefined GNRs was measured to be 60 ± 0.2 mV, which decreased to 42 ± 0.1 mV after removal of excess CTAB (two rounds of centrifugation and re-dispersion). A negative zeta potential of −30 ± 0.3 mV was measured after successful coating of the GNR surfaces with PSS. The positive zeta potential (40.3 ± 0.6 mV) of DOX-PSS-GNRs, due to the positive charge of DOX, confirmed the chemistry changes to the GNR surfaces (Figure 1d). Our results revealed a successful conjugation of DOX on the surface of PSS-GNRs with a higher stability in aqueous media than in other studies [18,30]. The percentage yield of the DOX-PSS-GNRs was found to be 81.2 ± 0.21 wt %.

### Drug loading efficiency

The loading efficacy of DOX on the PSS-GNRs was measured systematically using a standard curve of absorption of DOX (at 490 nm) by changing the concentration of DOX while keeping the concentration of PSS-GNRs constant (40 µg/mL). The drug loading capacity of PSS-GNRs was about 76% with a drug loading content of 3.2 µg DOX/mL of GNRs.

#### Photothermal stability of PSS-GNRs

**Figure 2:**
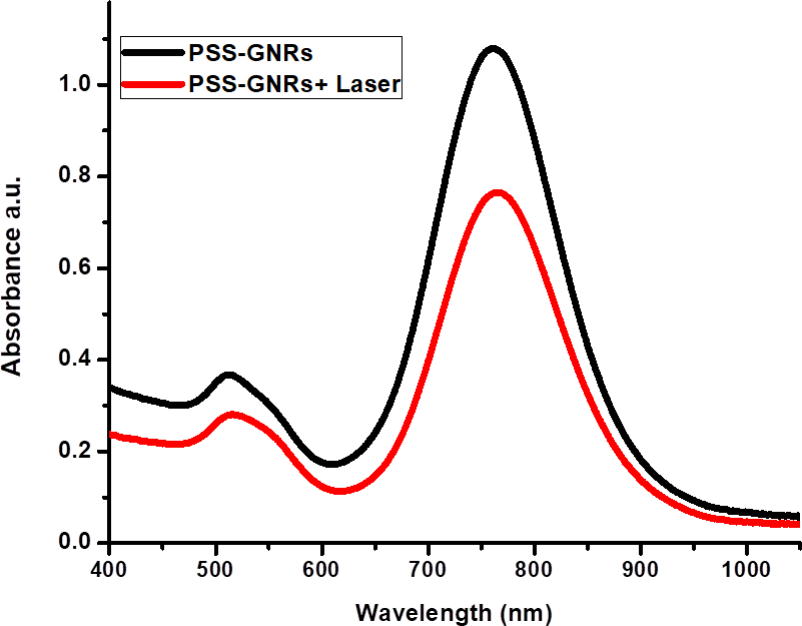
PSS-GNRs before and after 808 nm laser exposure, the LSPR peak shifted about 4 nm.

Optical characterization of PSS-GNRs showed that the LSPR peak of GNRs strongly depends on their aspect ratio. Hence, the LSPR peak position is an excellent indicator for any shape changes of GNRs. An aqueous solution of PSS-GNRs after laser exposure for 2 min (power density = 1.5 W/cm^2^) remained stable. The LSPR peak shifted by approximately 4 nm (Figure 2). The stability of PSS-GNRs after NIR laser exposure was sufficient for photothermal therapy.

#### In vitro DOX release after NIR irradiation

**Figure 3:**
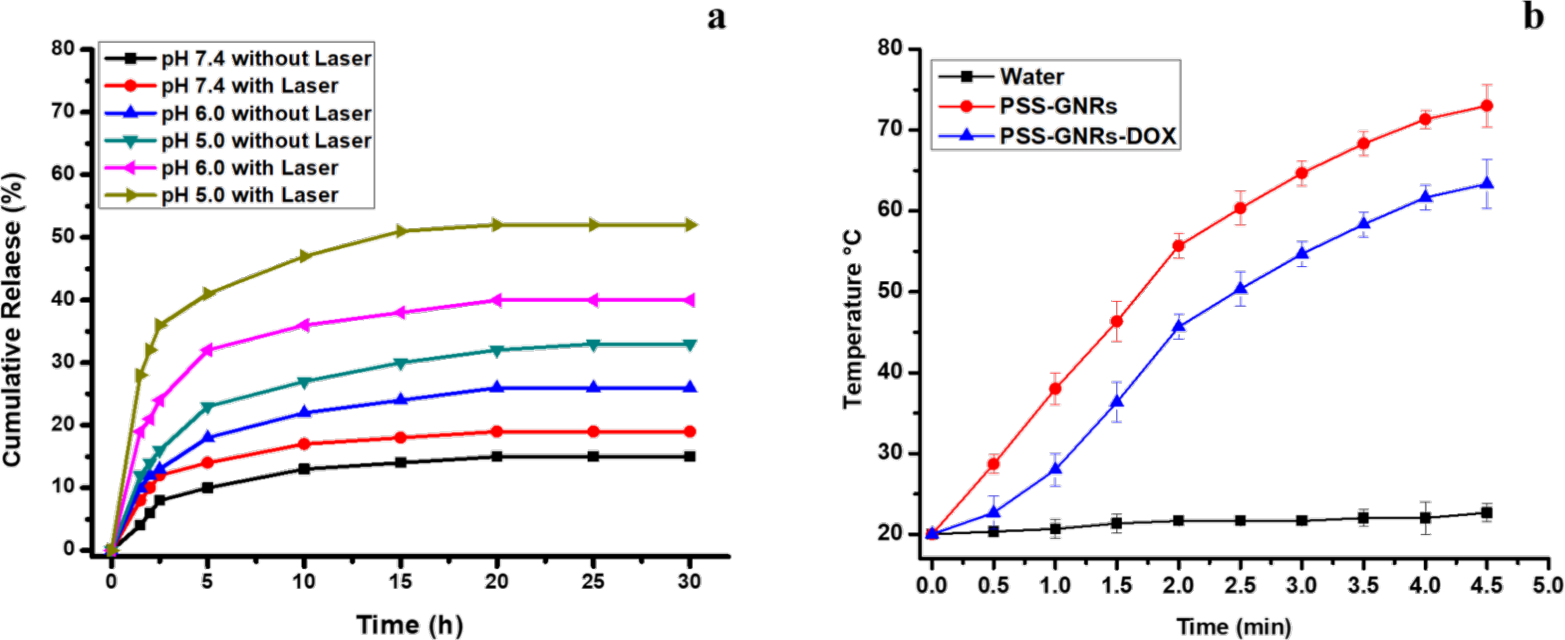
In vitro DOX release profile from PSS-GNRs. (a) NIR-triggered DOX release at different pH values. Fluorescence intensity was measured from 0 to 30 h. (b) Heating curves of water, PSS GNRs and PSS-GNRs-DOX (10 µg Au/mL) under NIR (808 nm) laser irradiation.

Drug release from PSS-GNRs can be easily controlled with NIR laser irradiation. The cumulative DOX release almost doubled after laser exposure (1.5 W/cm^2^) compared to non-irradiated samples (Figure 3). Enhanced drug release stimulated by laser (808 nm) may be related to the heat generated by the nanomaterial. Almost 40% of DOX was released at pH 5 from the laser-irradiated sample, compared to 22% from the non-irradiated sample at the same pH after 5 h (Figure 3). DOX release was reduced during the subsequent hour of incubation. The data showed that 50% of conjugated DOX was released from PSS-GNRs over a period of 30 h at pH 5. The microenvironment of the tumor cells could facilitate enhanced drug release due to the acidic pH value (approximately pH 5) of intracellular lysosomes and extracellular tissues of tumors [31].

In order to prove the photothermal conversion ability of the nanorods PSS-GNRS and PSS-GNRs-DOX (10 µg Au/mL) were exposed to NIR laser irradiation (808 nm) at a power density of 1.5 W/cm^2^ for 2 min. There was an increase in temperature to 52 °C and 45 °C, respectively. In contrast, no significant change in temperature was observed when water was exposed to the same laser irradiation (Figure 3b). This confirmed the light–heat transformation through the GNRs. This hyperthermic effect mediated by GNRs may be responsible for the laser-triggered release of DOX. These findings are consistent with previous studies [23].

#### PSS-GNRs nanocomplex biocompatibility

**Figure 4:**
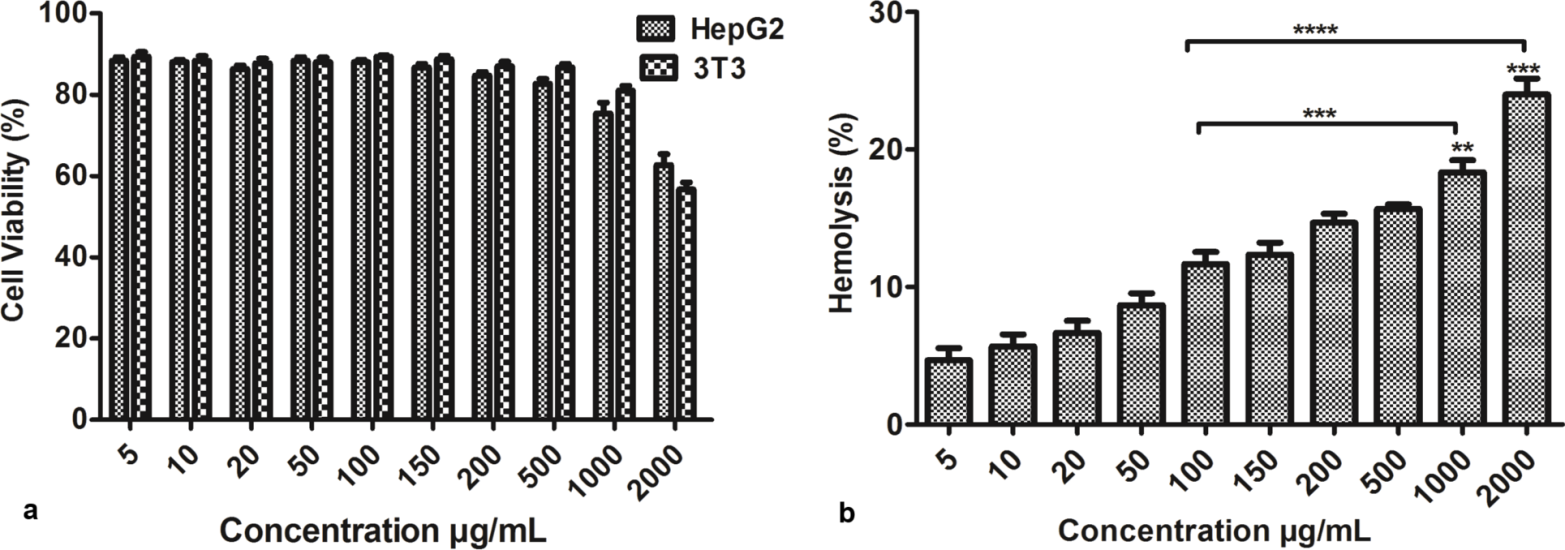
(a) Relative viabilities of HepG2 and 3T3 cells after being incubated with different concentrations of PSS-GNRs for 24 h. (b) A hemotoxicity assay on PSS-GNRs shows that PSS-GNRs are hemo-compatible. No significant difference was seen in the range of 5–500 µg/mL (<20% hemolysis). A low significant (***) of a high significant difference (****) were seen for 1000 and 2000 µg/mL compared with 100 µg/mL. Each bar shows the mean value ± SEM of triplicates.

Dose-dependent biocompatibility and cytotoxicity efficiency of the nanocarriers were measured in vitro. The efficiency of the GNRs in mediating cytotoxicity against HepG_2_ (carcinogenic) and 3T3 (non-carcinogenic) cells was evaluated. Cells were treated for 12 h with PSS-GNRs and analyzed using the MTT assay. As shown in Figure 4, cells treated with PSS-GNRs had no significant reduction in cell viability compared to control cells. The viability remained higher than 88% at concentrations of 500 µg/mL for HepG_2_ cells and 1000 µg/mL for 3T3 cells (Figure 4a). If nanoparticles interact with red blood cells (RBCs) in the blood stream they can cause hemolysis. Hemolytic properties and interaction with RBCs are the main parameters for the biocompatibility of nanocarriers [23]. Analysis of hemoglobin released from RBCs after incubation in a suspension of PSS-GNRs showed less than 20% hemolysis at a concentration of 1000 μg/mL (Figure 4b). The experiments revealed a good biocompatibility of PSS-GNRs, which was quantified by the concentration of hemoglobin in the supernatant of GNPs-RBCs mixture by monitoring absorbance intensity at 570 nm. The absence of a marked hemotoxicity of this sample is mainly related to the presence of the polymer. The GNR surface had no direct contact with the RBCs because it was completely passivated by the PSS coating [23].

#### Cell inhibition after NIR exposure of PSS-GNR-DOX complexes

**Figure 5:**
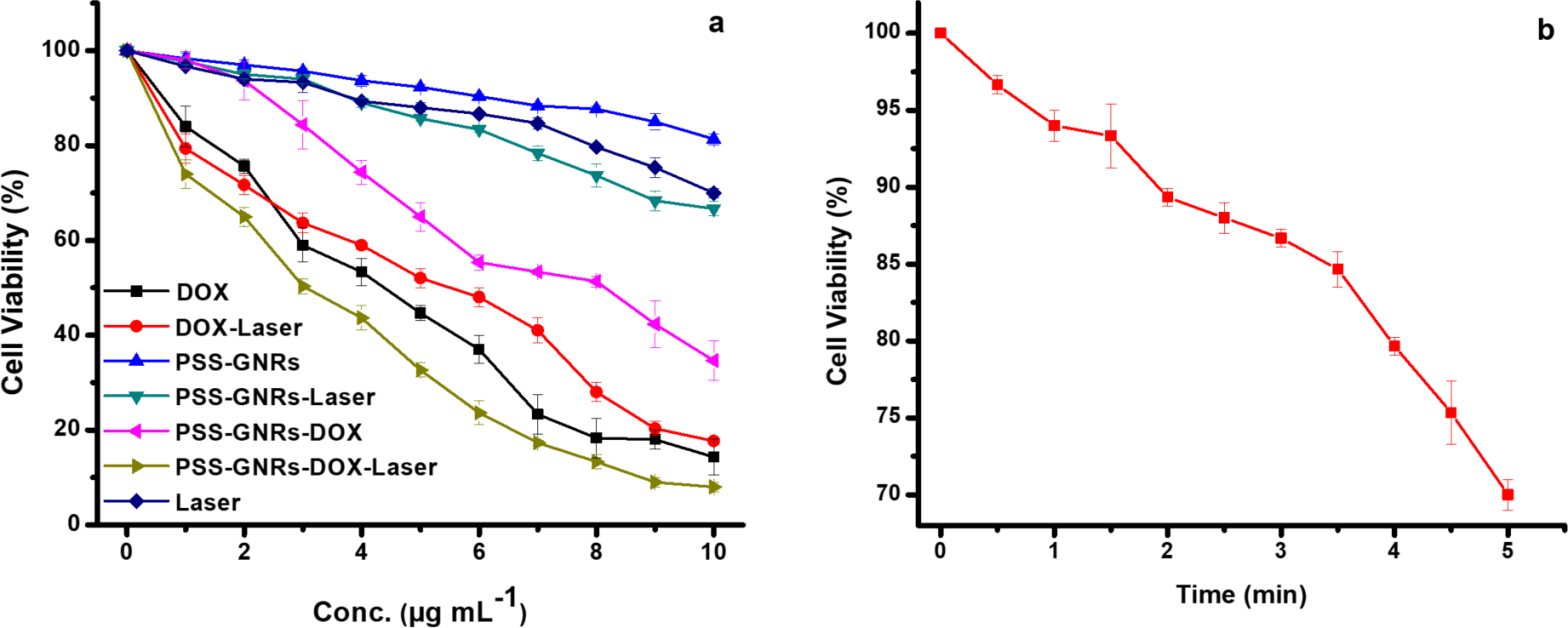
(a) Percentage viabilities of HepG2 cells treated with free DOX and DOX-PSS-GNRs exposed to NIR light (1.5 W/cm^2^ for 2 min per treatment, three treatments over 2 h). The cytotoxicity values with and without laser irradiation are significantly different with *p* < 0.05 in the case of DOX-PSS-GNRs according to a two-sample student *t*-test. (b) Percentage viabilities of HepG2 cells treated with NIR light (1.5 W/cm^2^) at different points in time.

Drug release from PSS-GNR-DOX triggered by NIR laser irradiation (808 nm) at an output power density of 1.5 W/cm^2^ with a beam spot size of 6 mm in diameter on HepG_2_ cells was studied. DOX release from PSS-GNR-DOX was increased significantly (*p* < 0.05) after 2 min of NIR irradiation (Figure 5). HepG_2_ cells were treated with free DOX and DOX-PSS-GNRs, either irradiated with NIR laser or not exposed to NIR light. A dose-dependent cytotoxicity was observed in all study groups. About 84% of cells were killed by free DOX and 65% by DOX-PSS-GNRs at an equivalent DOX concentration of 10 μg/mL (Figure 5). This showed that free DOX was more toxic than DOX conjugated to a nanocarrier at the same drug concentration. Similar findings were reported by other studies [32–33]. The high cytotoxic effect of free DOX is due to the higher availability of the drug to the cells after cell uptake. The decreased cytotoxicity of DOX-PSS-GNRs is because of a delayed drug release inside cells [23]. The PSS-GNRs nanocomplex shows potential as biocompatible nanocarrier for drug loading and delivery in cancer therapy.

Arunkumar et al. have reported that DOX-conjugated gold nanorods are highly biocompatible vehicles for sustained drug delivery, reduce cardiotoxicity in vivo, and have high photothermal efficacy [34]. A previous report showed that DOX-loaded tiopronin-coated gold nanoparticles (Au-TIOP-DOX) had a better efficacy in killing cancer cells than free DOX [35]. Similarly, a study showed an improved toxicity of DOX-loaded DNA-wrapped gold nanoparticles in drug-resistant cancer cells [36]. Our results are opposite to this study, it might be due to the higher sensitivity of HepG_2_ cells to DOX, which could induce more toxicity of free drug compared to conjugated one. The cytotoxic efficiency of the DOX-loaded PAA-PEG-GNRs was found to be similar to free DOX and improved with an increase in their concentrations [36]. In a previous study, cell viability was significantly decreased down to 57% using GNR-DOX-cRGD, whereas free DOX demonstrated the highest level of cytotoxicity (41% of control) in U87MG cells [35]. We found that DOX-PSS-GNRs complexes killed more cancer cells (93%) after NIR laser irradiation (Figure 5). The higher cytotoxicity of the complex is due to the enhanced drug release upon NIR laser irradiation. The IC_50_ value of PSS-GNRs-DOX was 7.99 ± 0.0032 µg/mL. For PSS-GNRs-DOX with laser irradiation it was 3.12 ± 0.0906 µg/mL. The IC_50_ values of free DOX and DOX with laser exposure were 3.999 ± 0.04211 and 4.41 ± 0.0037 µg/mL, respectively. Previously, Au-HNS-EGFR-DOX were reported to have a significant antiproliferative activity against lung cancer cells when irradiated with NIR laser (125 mW/cm^2^, 25 s), in contrast to non-irradiated cells [37]. Free DOX showed no significant influence on viability, neither with nor without laser irradiation. This indicates that increased cell death upon NIR laser irradiation might be attributed to the presence of the gold nanocarrier. Without laser treatment low drug release from the nanocomplex was observed. Laser-triggered DOX release was measured using the same laser treatment at different time intervals (2, 3, and 4 h) in which drug release was improved in a time-dependent manner. Less than 10% of DOX was released within 4 h from PSS-GNR-DOX without NIR irradiation under the same experimental conditions (Figure 5). Drug release from the nanocomplex (PSS-GNR-DOX) might be easily turned “on” and “off” by NIR laser exposure. The NIR laser irradiation causes a melting of PSS that would lead to decreased stability and an enhanced drug diffusion coefficient. No drastic change in temperature of the solution was observed after NIR irradiation. Hong et al. developed a system to estimate the photothermal conversion efficacy of GNRs for different irradiation laser powers and reported that exposure with 40 W/cm^2^ for 30 min generated heat on PEGylated GNRs necessary for photothermal ablation of MDA-MB-231 [38]. To minimize the thermotoxicity associated with laser exposure, in the current study, we used a low laser power density of 1.5 W/cm^2^, a shorter time of NIR irradiation (2 min), and a DOX concentration of 10 µg/mL. Under these conditions, we observed significant cell death (93%). Contrary to this, about 73% cell death at a higher laser power (2.5 W/cm^2^) with a longer exposure time of 5 min is reported by Liao et al. [23]. Similarly, in other study 74% cell death was reported using a low laser power density of 1.8 W/cm^2^ but with long exposure time and high drug concentration (20 µg/mL) [24]. Thus, we achieved a higher cell death rate at shorter exposure time and lower drug concentration.

## Conclusion

Multifunctional, biocompatible, and thermostable PSS-GNRs could be easily prepared by simple wet chemistry. A polymer was electrostatically conjugated, which facilitates the loading of DOX and its phototriggered release inside cancer cells in acidic environment. A comparatively good photothermal transfer ability has been achieved at a very low power density of 1.5 W/cm^2^ of NIR laser irradiation, as evidenced by the rapid temperature increase on the nanocarrier surface under 808 nm laser exposure for 2 min. A high cytotoxicity was observed with DOX-PSS-GNRs after NIR laser irradiation, in contrast to DOX alone. The GNRs could proficiently produce hyperthermia by converting NIR light into heat and kill heat-sensitive cancer cells with minimal side effects on the surrounding healthy cells due to the low power density of the laser and the shorter time of exposure. At the same time, the DOX release stimulated by the temperature rise could inhibit the proliferation of residual cancer cells.

The nanomaterial complex described here will have the capacity for cost-effective upscaling due to ease of synthesis and surface modification, and the tunable drug loading ability. Chemo-photothermal treatment based on nanocomplex systems is an efficient approach for reducing the high dose-related side effects in cancer management.

## Experimental

### Materials

CTAB (99.9%), hydrogen tetrachloroaurate(III) trihydrate (HAuCl_4_·3H_2_O 99%), ʟ-ascorbic acid (C_6_H_8_O_6_, 99%), sodium borohydride (NaBH_4_, 98%), silver nitrate (AgNO_3_, 99%), doxorubicin, (98%) poly(sodium 4-styrenesulfonate) (PSS; *M*_w_ = 70,000) and 3-(4,5-dimethylthiazol-2-yl)-2,5-diphenyltetrazolium bromide (MTT) were purchased from Sigma-Aldrich. Deionized (DI) water, having a resistance of 18 MΩ·cm, was used throughout the experiments.

### Gold nanorod synthesis

GNRs were synthesized through seed-mediated growth [25] with a slight modification [26]. Gold seed particles were synthesized by adding 250 μL of 10 mM HAuCl_4_·3H_2_O to 10 mL of 0.1 M CTAB under continuous stirring. 600 μL of freshly prepared, ice-cold NaBH_4_ solution (10 mM) was added followed by 30 min of continuous stirring. For the GNR growth solution, 50 mL of 0.1 M CTAB was added to 2.5 mL of 10 mM HAuCl_4_·3H_2_O. To the stirring solution 400 μL HCl (1 M), 500 µL AgNO_3_ (10 mM), and 400 μL ʟ-ascorbic acid (10 mM) was added. Finally, 200 μL of seed solution was added to the growth solution. GNRs were purified by centrifugation (14,000*g* for 20 min) after 24 h of incubation. Then, the collected pellet was re-dispersed in deionized water.

### PSS coating of GNRs

A reported method by Venkatesan et al. was used, with a slight modification, for the PSS coating of GNRs [18]. Prepared GNRs (2 mL, 40 µg/mL) were centrifuged at 12,000*g* for 10 min and the pellet was re-dispersed in 2 mL of deionized water. GNR solution was added drop-wise to 2 mL of PSS (2 mg/mL in 8 mM NaCl). For maximum adsorption, the solution was kept under stirring at room temperature for 2 h. Excess polymer (supernatant fraction) was removed by centrifugation (12,000*g* for 10 min). The PSS-stabilized GNRs were re-suspended in 2 mL deionized water and stored at 4 °C.

### Doxorubicin-loaded PSS-GNRs

The anticancer drug DOX was loaded onto the surface of PSS-GNRs by a previously reported simple stirring method with slight modifications [30]. PSS-GNRs (40 µg/mL, 2 mL) were added to an aqueous solution of DOX at a final concentration of 10 µg/mL and were stirred overnight in the dark at room temperature. Excess drug was removed by centrifugation at 12,000*g* for 10 min and the pellet was re-dispersed in 2 mL deionized water. UV–vis spectra of DOX-loaded GNRs were scanned at a wavelength range of 400–1100 nm. The surface charge distribution of DOX-loaded PSS-GNRs, at a different level, was determined by using a zeta potential analyzer (Zetasizer Nano ZS90 DLS system Malvern Instruments Ltd., England).

### Percentage yield

The nanoparticles were collected and weighed accurately. The percentage (%) yield was then calculated using the formula given below [39]:


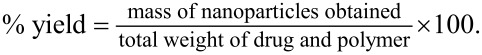


### Drug loading efficiency (DLE)

In order to calculate the drug loading efficiency, a known quantity of DOX was mixed with an aqueous PSS-GNRs solution (40 mg/mL) to get final drug concentrations of 5, 10, 15, 20, 25, 50, 100, 200, and 300 mg/mL. Then the suspension was stirred overnight in the dark at 20 °C. The suspension was then centrifuged at 12,000*g* for 10 min in order to precipitate the DOX-PSS-GNRs nanoconjugate and then dialyzed against pure water to remove unbound DOX by a previously described method [30]. The quantity of loaded DOX was measured at 485 nm. Drug loading efficiency (DLE) was calculated using the formula given below:


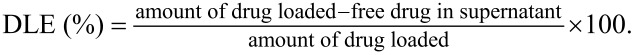


### Photothermal stability of PSS-GNRs

The photothermal stability of PSS-GNRs was measured using a previously described method [23]. Briefly, the aqueous solution of PSS-GNRs was irradiated with NIR laser (power density = 1.5 W/cm^2^) for 2 min and analyzed by UV–vis spectroscopy.

### In vitro drug release by NIR exposure

NIR-triggered drug release from PSS-GNRs was measured in 10 mM phosphate-buffered saline (PBS, pH 5.6 at 37 °C). A continuous-wave 808 nm NIR laser (Ti-Sapphire, Spectra Physics CA 95054, USA) was used. DOX-PSS-GNRs (40 µg/mL, 2 mL) were dispersed in 10 mL of PBS followed by NIR laser irradiation at an output power of 1.5 W/cm^2^ for 2 min and 800 μL of the solution was taken out for analysis. Exposed media was centrifuged at 12,000*g* for 10 min. The amount of DOX released from PSS-GNRs in the supernatant was determined by fluorescence measurements (Biotek synergy H4 multi-mode plate reader) following the method reported in [23].

### In vitro cytotoxicity assays

The in vitro cytotoxicity of PSS-GNRs was measured using 3T3 and HepG_2_ cells. Cells were seeded in 96-well plates (4 × 10^3^ cells per well) in 100 μL DMEM supplemented with 10% FBS and 1% pen–strep. After 24 h of incubation, cells were exposed to different concentrations of PSS-coated GNRs and were allowed to incubate at 37 °C for additional 24 h. Viability was measured by the MTT assay [40].

### Hemolysis assay

All human blood samples in this study were from healthy volunteers and used with Institutional Review Board (IRB) bioethics approval. The hemolysis assay was carried out according to the protocol from National Cancer Institute (NCI). Whole blood (5 mL) from two healthy human donors was drawn directly into K2-EDTA-coated tubes to prevent coagulation. Blood collection was performed by a trained phlebotomist in order to minimize the risk to the donor. A written informed consent was obtained from each donor prior to the blood drawn.

To the 5 mL of blood 15 mL of sterilized phosphate buffer saline (PBS) was added and, after slow agitation, tubes were centrifuged at 500*g* for 10 min. Supernatant containing plasma was aspirated and the buffy coat was washed thrice and diluted with normal saline to a 50% packed cell volume (hematocrit) adjusted at pH 7.4 and stored at 4 °C. Different concentrations of PSS-GNRs (100 μL each) were incubated with 100 μL of RBCs suspension at 37 °C in CO_2_ incubator for 4 h. 0.2% Triton X-100 was used as positive control and PBS was taken as negative control [41]. After incubation, 50 μL of 2.5% glutaraldehyde was added to the sample in order to stop the process of hemolysis and centrifuged at 1000*g* for 10 min. Hemoglobin release was monitored at 562 nm using a microplate reader (Platos R496, Austria) by transferring supernatant to a 96-well plate. Percentage hemolysis was calculated using the following formula:


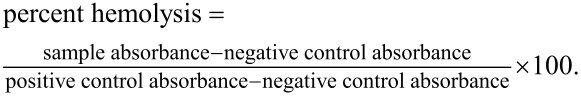


### Cell inhibition after photothermal treatment

Combination therapy was performed by the method described in a previous study with modifications [28]. The HepG_2_ cells were seeded into 96-well plates (5 × 10^3^ per well) and incubated for 24 h before the adding the different concentrations of PSS-GNRs, free DOX, and PSS-GNRs-DOX conjugate. The treated cells were incubated for 12 h for proper uptake before laser irradiation. After that, cells were illuminated with a 808 nm NIR laser (power density = 1.5 W/cm^2^ for 2 min) with a beam spot of 6 mm in diameter and incubated at 37 °C for 24 h. The MTT assay was performed to measure cell inhibition.
